# Effect of *N*-n-butyl haloperidol iodide on ROS/JNK/Egr-1 signaling in H9c2 cells after hypoxia/reoxygenation

**DOI:** 10.1038/srep11809

**Published:** 2015-07-02

**Authors:** Yanmei Zhang, Han Liao, Shuping Zhong, Fenfei Gao, Yicun Chen, Zhanqin Huang, Shishi Lu, Ting Sun, Bin Wang, Weiqiu Li, Han Xu, Fuchun Zheng, Ganggang Shi

**Affiliations:** 1 Department of Pharmacology, Shantou University Medical College, Shantou 515041, Guangdong, China; 2 Department of Biochemistry and Molecular Biology, University of Southern California, Los Angeles, California 90033, USA; 3 Analytical Cytology Laboratory, Shantou University Medical College, Shantou 515041, Guangdong, China; 4 Department of Pharmacy, the First Affiliated Hospital, Shantou University Medical College, Shantou 515041, Guangdong, China; 5 Department of Cardiovascular Diseases, the First Affiliated Hospital, Shantou University Medical College, Shantou 515041, Guangdong, China

## Abstract

Reactive oxygen species (ROS)-induced oxidative stress in cells is an important pathophysiological process during myocardial ischemia/reperfusion (I/R) injury, and the transcription factor Egr-1 is a master switch for various damage pathways during reperfusion injury. An *in vitro* model of myocardial I/R injury and H9c2 cardiomyoblast cells hypoxia/reoxygenation (H/R) was used to assess whether there is abnormal intracellular ROS/JNK/Egr-1 signaling. We also assessed whether *N*-n-butyl haloperidol (F_2_), which exerts protective effects during myocardial I/R injury, can modulate this pathway. H/R induced ROS generation, JNK activation, and increased the expression of Egr-1 protein in H9c2 cells. The ROS scavengers edaravone (EDA) and N-acetyl-L-cysteine (NAC) reduced ROS level, downregulated JNK activation, and Egr-1 expression in H9c2 cells after H/R. The JNK inhibitor SP600125 inhibited Egr-1 overexpression in H9c2 cells caused by H/R. F_2_ could downregulate H/R-induced ROS level, JNK activation, and Egr-1 expression in H9c2 cells in a dose-dependent manner. The ROS donor hypoxanthine-xanthine oxidase (XO/HX) and the JNK activator ANISO antagonized the effects of F_2_. Therefore, H/R activates ROS/Egr-1 signaling pathway in H9c2 cells, and JNK activation plays an important role in this pathway. F_2_ regulates H/R-induced ROS/JNK/Egr-1 signaling, which might be an important mechanism by which it antagonizes myocardial I/R injury.

Myocardial ischemia/reperfusion (I/R) injury often occurs with angioplasty, thrombolytic therapy, ischemic heart disease, and cardiac surgery. However, its pathogenesis remains unclear. A large number of studies have shown that reactive oxygen species (ROS)-induced oxidative stress (tissue damage caused by the accumulation of ROS in cells) is an important pathophysiological process during myocardial I/R injury^1^. ROS are a class of chemically reactive metabolites that contain oxygen, including superoxide anion (O_2_^•−^), hydroxyl radical (·OH), and hydrogen peroxide (H_2_O_2_)^2^. An appropriate amount of ROS usually has a cardioprotective effect; however, excessive ROS not only directly and non-specifically oxidize biological macromolecules such as DNA, proteins, and lipids, but also damage cells by activating a redox signaling cascade that ultimately leads to cell death^3^,^4^. In recent years, ROS have generally been described as second messengers because of their key function in cellular signaling cascades and pathophysiological processes such as proliferation, gene expression, adhesion, differentiation, senescence, apoptosis, and necrosis^4^,^5^,^6^.

Early growth response gene -1 (Egr-1) is a transcription factor that transmits information across the nuclear membrane. It can be rapidly induced and then initiates a series of downstream target genes in all cell types, therefore, it is also known as third messengers^7^,^8^. Many studies have suggested that Egr-1 is a master switch for various pathways of reperfusion injury^9^. Our early *in vitro* and *in vivo* studies demonstrated that Egr-1 overexpression is an important cause of myocardial ischemia-reperfusion injury^10^,^11^.

Because ROS and Egr-1 have an obvious sequential relationship in intracellular signaling pathways and Egr-1 is considered to be I/R’s damage hub, it is possible that ROS and Egr-1 also have a sequential relationship during I/R. For example, Egr-1 might act as the “damage hub” to mediate oxidative stress and the related ROS-induced damage during I/R.

*N*-butyl haloperidol iodide (F_2_) is a novel compound synthesized by our laboratory (Chinese national invention patent, No. ZL96119098.1). Extensive research studying the efficacy and mechanism of action of F_2_ revealed that F_2_ could improve I/R or hypoxia/reoxygenation (H/R)-induced myocardial tissue or myocardial cell damage by blocking membrane L-type Ca^2^^+^ channels and inhibiting Egr-1 overexpression. Classic calcium antagonists also inhibit the expression of Egr-1, suggesting that the F_2_-mediated inhibition of Egr-1 expression might be related to its inhibition of Ca^2+^ 10,12,13,14. In addition, we found that F_2_ could antagonize I/R- or H/R-induced decreased SOD activity and increased malondialdehyde (MDA; an oxidative stress product) level, which suggested that F_2_ might play a negative regulatory role in abnormal ROS generation during myocardial I/R or H/R^15^. Ca^2+^ and ROS are related; for example, Ca^2+^ in cytoplasm when reperfusion can be transported to the mitochondrial matrix, reducing ATP synthesis and increasing ROS generation. Therefore, it could be speculated that high levels of ROS might lead to the overexpression of Egr-1 during myocardial I/R. As such, F_2_ might antagonize myocardial I/R-associated damage by decreasing the intracellular Ca^2+^ overload and the subsequent generation of ROS and overexpression of Egr-1.

Mitogen-activated protein kinases (MAPKs) are a protein kinase family that can phosphorylate both serine and tyrosine residues. The classic MAPK signaling pathways include extracellular signal-regulated kinase (ERK1/2), c-Jun N-terminal kinase (JNK), and p38 mitogen-activated protein kinase (p38 MAPK). JNK is an important intracellular stress regulatory protein. Many studies have demonstrated that increased levels of oxidative stress/ROS activate JNK signaling, which can increase the incidence of diseases and/or the development of aging-related neurodegenerative diseases, ischemia-reperfusion injury and diabetes^16^,^17^,^18^. JNK might be the upstream signal that induces Erg-1 expression^19^; therefore, JNK might mediate the signaling between ROS and Egr-1 during myocardial I/R. In the current study, an H9c2 cardiomyoblast cell line-based H/R model was used to determine whether ROS/JNK/Egr-1 signaling pathways were active in H9c2 cells after H/R and to determine if F_2_ exerted any protective effects against myocardial I/R injury via these pathways.

## Results

### ROS levels and Egr-1 protein expression in H9c2 cells during H/R over time

**Figure 1:**
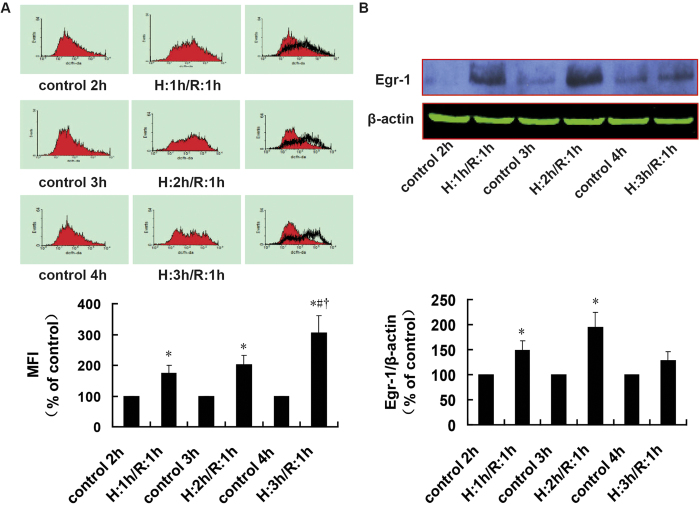
ROS levels and Egr-1 protein expression in H9c2 cells with different durations of H/R, as assessed using flow cytometry and western blotting. A. ROS levels during H/R; *n *= 6. B. Cropped blots show protein levels of Egr-1 and β-actin; *n *= 4. The bands were excised from the same gel. Data are expressed as the percentages of the control group. All values are expressed as mean ± S.E.M. ^*^P < 0.05 vs. control; ^#^*P *< 0.05 vs. the H:1 h/R:1 h group; ^†^*P *< 0.05 vs. the H:2 h/R:1 h group.

As shown in Fig. 1A, ROS levels were significantly higher in H9c2 cells than in control groups at each time point (*P *= 0.004, *P *= 0.001, and *P *= 0.004). In addition, the effects were time-dependent and increased with longer H/R times. Therefore there was a significant difference between the ROS levels in the H: 3 h/R: 1 h group and H: 1 h/R: 1 h or H: 2 h/R: 1 h groups (*P *= 0.010 and *P *= 0.015).

As shown in Fig. 1B, compared with the control groups, the expression of Egr-1 increased in the H: 1 h/R: 1 h group, H: 2 h/R: 1 h group and H: 3 h/R: 1 h groups, whereas the increase in the H: 1 h/R: 1 h and H: 2 h/R: 1 h groups was significant (*P *= 0.006 and *P *= 0.024). However, the peak expression fell in the H: 2 h/R: 1 h group. Based on the above data, subsequent experiments were performed using 2 h of hypoxia and 1 h of reoxygenation.

### Relationship between ROS levels and Egr-1 expression during H/R

**Figure 2:**
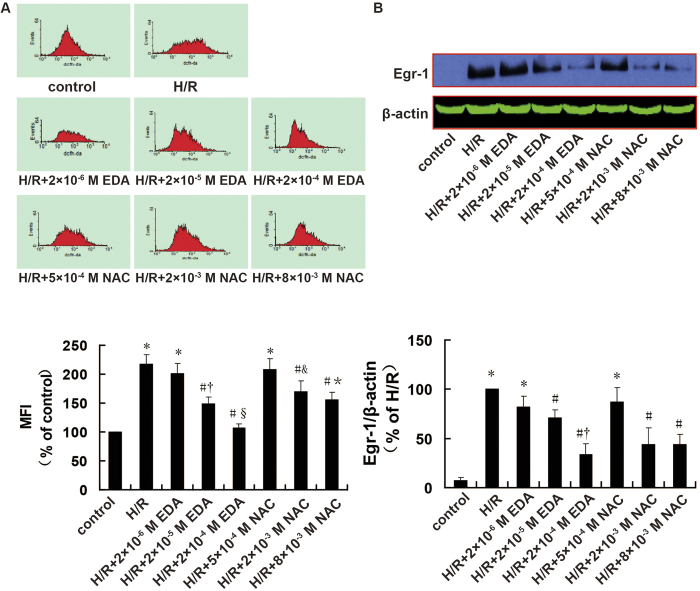
Effects of different doses of ROS scavengers on ROS level and Egr-1 protein expression in H9c2 cells after H/R, as assessed using flow cytometry and western blotting. A. ROS levels; *n *= 6. B. Cropped blots show protein levels of Egr-1 and β-actin; *n *= 3. The bands were excised from the same gel. Data are expressed as the percentages of the control or H/R groups. All values are expressed as mean ± S.E.M. ^*^P < 0.05 vs. control; ^#^*P *< 0.05 vs. H/R; ^†^*P *< 0.05 vs. H/R + 2 × 10^−6^ M EDA; ^§^*P *< 0.05 vs. H/R + 2 × 10^−5^ M EDA; ^&^*P *< 0.05 vs. H/R + 5 × 10^−4^ M NAC; ^*^P < 0.05 vs. H/R + 2 × 10^−3^ M NAC.

The changes in ROS level and Egr-1 expression in H9c2 cells are shown in Fig. 2. Compared with the control group, ROS levels and Egr-1 expression were increased in the H/R groups significantly (*P *< 0.001 and *P *< 0.001). Compared with the H/R groups, the ROS scavengers edaravone (EDA) and N-acetyl-L-cysteine (NAC) decreased ROS level and Egr-1 expression in H9c2 cells in a dose-dependent manner. Moderate and high concentrations of ROS scavengers (2 × 10^−5^ M EDA, 2 × 10^−4^ M EDA, 2 × 10^−3^ M NAC, and 8 × 10^−3^ M NAC) resulted in substantial decreases in H/R-induced ROS level (*P *= 0.003, *P *< 0.001, *P *= 0.011 and *P *= 0.002) and Egr-1 expression (*P *= 0.027, *P *= 0.001, *P *= 0.027 and *P *= 0.006). In contrast, low concentrations of ROS scavengers (2 × 10^−6^ M EDA, 5 × 10^−4^ M NAC) had no significant effect on ROS levels (*P *= 0.235 and *P *= 0.370) or Egr-1 expression induced by H/R (*P *= 0.233 and *P*=0.102). Linear correlation analysis between ROS level and Egr-1 expression in H9c2 cells revealed that they were positively correlated (*r *= 0.91, *P *= 0.002).

### Relationship between ROS and JNK

Xanthine oxidase (XO) is an important oxidase for ROS formation, and it releases a large number of electrons by catalyzing hypoxanthine (HX) to form xanthine (X) and uric acid. The electrons are accepted by oxygen generating O_2_^•−^, which rapidly disproportionates into H_2_O_2_. Both HX and X are XO substrates that generate ROS; therefore, XO/X and XO/HX mixtures can be used as ROS generating agents^20^,^21^,^22^. In the current study we used XO/HX to increase ROS levels and EDA and NAC to decrease ROS levels to assess if JNK activation or the upregulation of Egr-1 expression are mediated by ROS.

**Figure 3:**
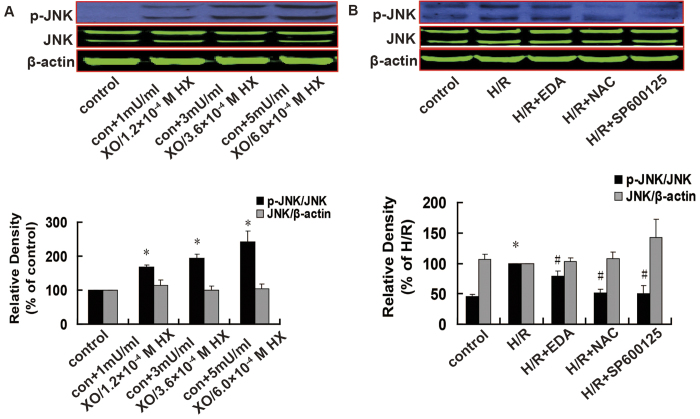
Effects of a ROS donor, ROS scavengers, and a JNK inhibitor on the levels of total and p-JNK expression in H9c2 cells, as assessed using western blotting. A. Effects of a ROS donor; *n *= 6. B. Effects of ROS scavengers and a JNK inhibitor. *n *= 4. Cropped blots show protein levels of p-JNK, total JNK and β-actin. The bands were excised from different gels which were run under the same electrophoresis condition. Data are expressed as percentages of the control or H/R groups. All values are presented as mean ± S.E.M. ^*^P < 0.05 vs. control; ^#^*P *< 0.05 vs. H/R.

Compared with control, JNK activation was increased substantial in the XO/HX groups in a dose-dependent manner, whereas total JNK expression was unchanged (Fig. 3A). Conversely, JNK activation was decreased significantly in the presence of the ROS scavengers EDA (*P *= 0.027) and NAC (*P *< 0.001) and JNK inhibitor (*P *= 0.006), but total JNK expression was unchanged (Fig. 3B).

### Relationship between JNK and Egr-1

**Figure 4:**
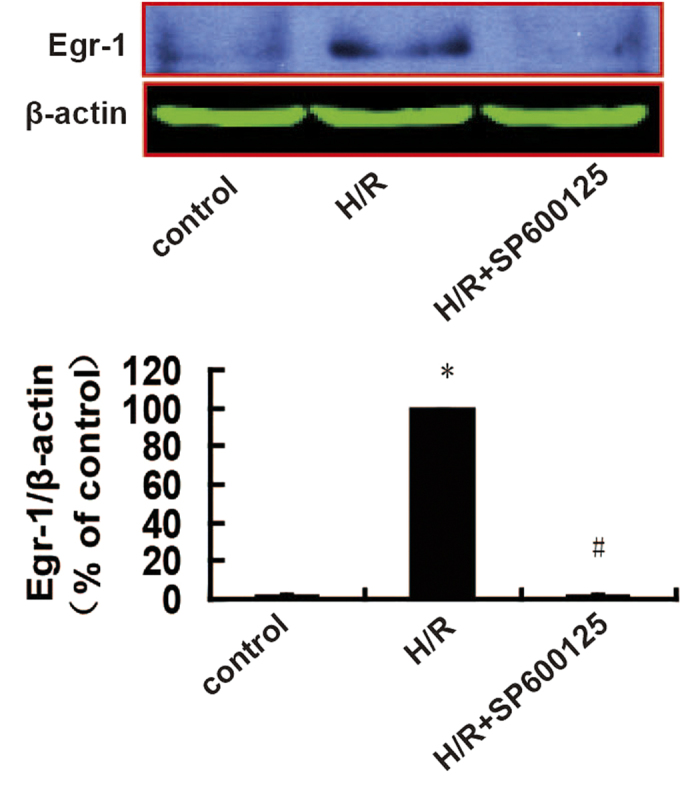
Effects of a JNK inhibitor on Egr-1 expression in H9c2 cells after H/R using western blotting. Cropped blots show protein levels of Egr-1 and β-actin. The bands were excised from the same gel. Data are expressed as percentages of the H/R group. All values are presented as mean ± S.E.M; *n *= 4. ^*^P < 0.05 vs. control; ^#^*P *< 0.05 vs. H/R.

We used the JNK inhibitor SP600125 determine which of JNK or Egr-1 was upstream from the other. As shown in Fig. 4, compared with the control group, Egr-1 expression in H9c2 cells was increased significantly in the H/R group (*P *< 0.001), which was inhibited by the JNK inhibitor (*P *= 0.02).

### Effect of F_2_ on ROS level, JNK activation, and Egr-1 expression

**Figure 5:**
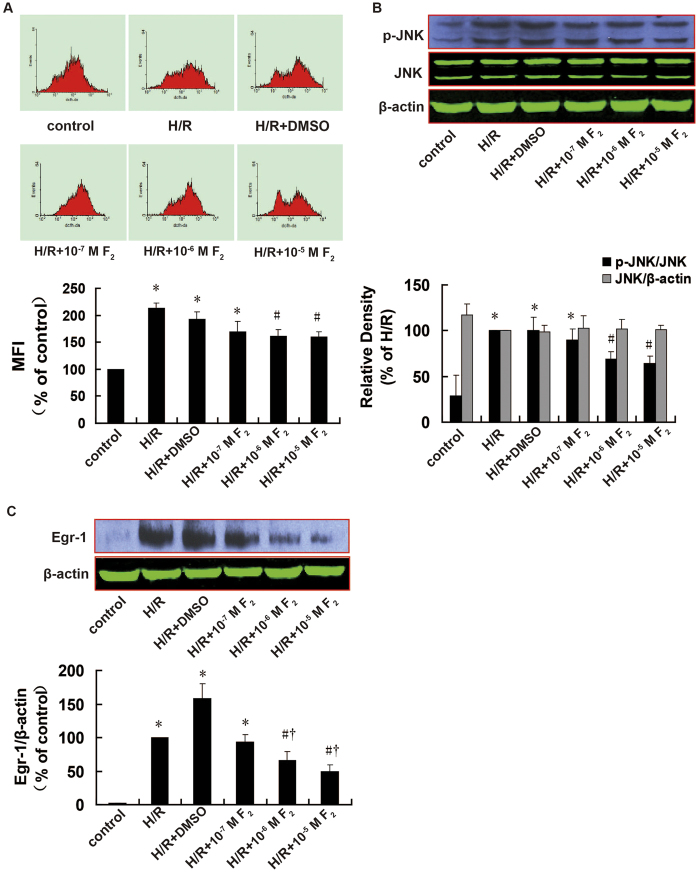
Effects of F_2_ on ROS levels, JNK activation, and Egr-1 expression in H9c2 cells after H/R, as assessed using flow cytometry and western blotting. **A**. ROS levels; *n *= 10. **B**. Cropped blots show total and p-JNK expressions; *n *= 6. The bands were excised from different gels which were run under the same electrophoresis condition. **C**. Cropped blots show Egr-1 and β-actin expressions; *n *= 6. The bands were excised from the same gel. Data are expressed as percentages of the levels of the control or H/R groups. All values are expressed as mean ± S.E.M. ^*^P < 0.05 vs. control; ^#^*P *< 0.05 vs. H/R; ^†^*P *< 0.05 vs. H/R + 10^−7^ M F_2_.

After confirming the activation of the ROS/JNK/Egr-1 signaling pathway in H9c2 cells during H/R, we next explored the effects of F_2_ on this pathway. As shown in Fig. 5, compared with control, ROS level (*P *< 0.001), JNK activation (*P *< 0.001), and Egr-1 expression (*P *< 0.001) increased significantly in the H/R group; however, these effects were inhibited by F_2_ in a dose-dependent manner (1 × 10^−7^ M, 1 × 10^−6^ M, 1 × 10^−5^ M). Compared with the H/R group, ROS levels (*P *= 0.021 and *P *= 0.006), JNK activation (*P *= 0.002 and *P *=0.002), and Egr-1 expression (*P *= 0.031 and *P *= 0.015) were significantly lower in the presence of moderate and high F_2_ concentrations (1 × 10^−6^ M, 1 × 10^−5^ M). In addition, moderate and high F_2_ concentrations had significantly stronger effects on Egr-1 protein expression compared with low concentrations (*P *= 0.048 and *P *= 0.016). These data suggest that F_2_ can inhibit H/R-induced ROS high-level, JNK activation, and Egr-1 expression in H9c2 cells in a dose-dependent manner. There were no differences in any measurements between the H/R and H/R+dimethyl sulfoxide (DMSO) groups, indicating that the solvent did not affect the results. There was also no change in total JNK expression among groups (*P *> 0.05).

### ROS/JNK/Egr-1 agonists antagonize the effects of F_2_

**Figure 6:**
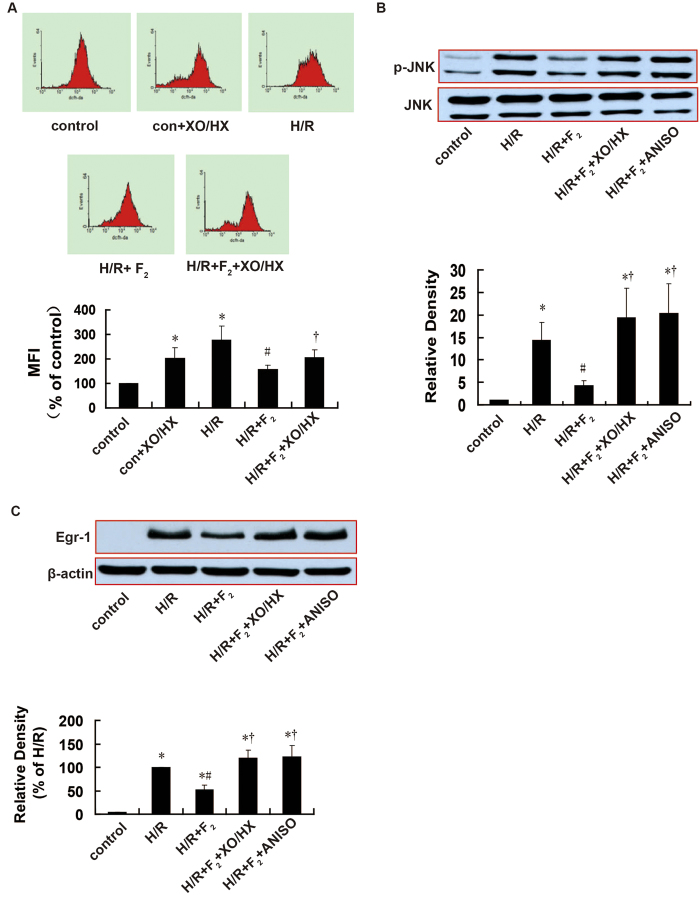
Influence of a ROS donor and JNK activator on the effects of F_2_ on ROS level, JNK activation, and Egr-1 expression in H9c2 cells after H/R, as assessed using flow cytometry and western blotting. **A**. ROS levels; *n *= 6. **B**. Cropped blots show total and p-JNK expressions; *n *= 3. The bands were excised from different gels which were run under the same electrophoresis condition. **C**. Cropped blots show Egr-1 and β-actin expressions; *n *= 3. The bands were excised from the same gel. Data are expressed as percentages of the levels of the control or H/R groups. All values are expressed as means ± S.E.M. ^*^P < 0.05 vs. control; ^#^*P *< 0.05 vs. H/R; ^†^*P *< 0.05 vs. H/R + 10^−6^ M F_2_.

As shown in Fig. 6, the ROS donor XO/HX antagonized the effects of F_2_ on H/R-induced ROS generation (*P = *0.021), JNK activation (*P = *0.045), and Egr-1 expression (*P = *0.014). Similarly, the JNK activator anisomycin (ANISO) antagonized the effects of F_2_ on H/R-induced JNK activation (*P = *0.037) and Egr-1 overexpression (*P = *0.027).

## Discussion

It is widely accepted that oxidase stress caused by ROS accumulation, calcium overload, and inflammation are important pathophysiological changes and therapeutic targets for myocardial I/R injury. In recent years, studies analyzing the signaling pathways involved in I/R injury have provided new targets for myocardial I/R injury prevention and therapy. It was suggested that calcium and ROS are second messengers during I/R, functioning to transmit information from the cell membrane to the cytoplasm. Transcription factors function as third messengers to further transmit the information to the nucleus, binding to nuclear DNA and altering downstream target gene expression, which eventually leads to I/R injury.

Egr-1 is an immediate-early gene, and a number of stimuli such as hypoxia and mechanical damage can result in its expression. It can bind to specific DNA sequences, triggering downstream target gene expression. In the year 2000, Yan *et al.*^9^ reported that Egr-1 plays roles in three pathological changes during I/R injury: inflammation, coagulation, and high vascular permeability. They subsequently proposed that Egr-1 has a central and unifying role in the pathogenesis of ischemic tissue damage^9^. Consistent with this, we previously used antisense oligonucleotides in the myocardial I/R model *in vivo* and *in vitro* to demonstrate that Egr-1 expression is an important cause of myocardial I/R injury^11^.

In recent years, studies revealed that a variety of stimuli such as drugs or glucose deprivation could activate ROS/Egr-1 pathways in different cells^23^,^24^,^25^,^26^. Aggeli *et al.*^27^ and Hartney *et al.*^28^ reported that Egr-1 expression could be induced by ROS-generating enzymes such as XO or by direct H_2_O_2_ treatment. In addition, Nozik-Grayck *et al.*^29^ found that hypoxia-induced upregulation of Egr-1 and its downstream target protein were inhibited in transgenic mice overexpressing extracellular SOD (EC-SOD). This might be related to the direct elimination of ROS by EC-SOD. Taken together, these studies demonstrated that ROS positively regulate Egr-1. Although it is known that oxidative stress and Egr-1 play roles in myocardial I/R injury, it remains unclear if there is a direct relationship between ROS (the second messenger) and Egr-1 (the third messenger) during myocardial I/R.

In the current study, H9c2 cardiomyoblasts was used as our experimental setting. This cell line derived from embryonic heart ventricle has been extensively used in studies investigating signal transduction mechanisms in cardiomyocytes because it retains properties of signaling pathways of cardiomyocytes. We used different doses of the ROS scavengers EDA and NAC during H9c2 cells H/R to demonstrate dose-dependent effects on ROS levels and Egr-1 expression. Both ROS scavengers could decrease H/R-induced high concentrations of ROS and Egr-1 overexpression. Intracellular ROS level and Egr-1 expression were highly positively correlated. The limited conditions of the experiment prevented us from detecting ROS levels and Egr-1 expression simultaneously within the same cell or group of cells. Nevertheless, we ensured that other non-experimental factors were consistent among groups (the growth state of the cells, the density of cultured cells, drug treatment, and the timing of cell synchronization). Therefore, the correlation between ROS level and Egr-1 expression is convincing, and suggests that intracellular ROS levels modulate Egr-1 expression. This supports the hypothesis that abnormal ROS/Egr-1 signaling occurs during H9c2 cells H/R. Mo *et al.*^30^ revealed that ambient ultrafine particles (UFPs) and/or cigarette smoke extract (CSE) activated NADPH oxidase, resulting in ROS generation, activation of the MAPKs p38 and ERK1/2, and the upregulation of Egr-1 in mouse pulmonary microvascular endothelial cells. Aggeli *et al.*^27^ reported that exogenous ROS (H_2_O_2_) could stimulate Egr-1 expression and nuclear translocation via ERK or JNK in H9c2 cells. The current study revealed that ROS positively regulate Egr-1 in a H9c2 cell H/R model. In addition, ROS scavengers EDA and NAC and the JNK inhibitor SP600125 were used to determine whether JNK intervenes in ROS/Egr-1 signaling. The results revealed that ROS scavengers inhibited H/R-induced JNK activation and Egr-1 overexpression substantially. Therefore, JNK and Egr-1 are downstream signaling of ROS. The JNK inhibitor SP600125 downregulated H/R-induced Egr-1 expression, suggesting that JNK is upstream signaling of Egr-1. There was no considerable difference in total JNK expression among groups, indicating that no new JNK protein was expressed during H9c2 cell H/R. These results demonstrate that Egr-1, a transcription factor sensitive to redox regulation^2^, can be activated by high concentrations of ROS induced by various stimuli. However, the signal-mediating MAPK can be quite different due to different cell types or pathological models. In conclusion, the current study revealed that H/R stimulus could cause ROS generation, which activated JNK, increased the expression of Egr-1, and subsequently caused H/R injury. Therefore, the ROS/JNK/Egr-1 signaling pathway mediates H9c2 cell H/R injury. These results also demonstrated that ROS-mediated oxidative stress-related damage in myocardial I/R is also associated with Egr-1, and that Egr-1 plays a key role in myocardial I/R injury pathway. In this way, this study provides a further explanation for Egr-1, acting as a master switch in I/R injury, a view proposed by Yan *et al.*^9^.

It has been demonstrated previously that F_2_ is a novel calcium antagonist that can block L-type calcium channel in the cardiomyocyte membrane, reduce Egr-1 gene and protein expressions, and antagonize myocardial I/R and H/R injury. A previous study revealed that classical calcium antagonists such as verapamil, diltiazem, and nifedipine significantly reduced Egr-1 overexpression induced by I/R in cardiac tissue and H/R in myocardial cells^15^. The data show that regulating Egr-1 expression is another mechanism that, like regulating calcium channel, calcium antagonists protect I/R cardiac tissues and H/R myocardial cells. This is consistent with the hypothesis that increased calcium concentrations are a prerequisite for increasing the expression of *Egr-1*^31^. ROS-induced oxidative stress and Ca^2+^ overload are the two main causes of myocardial I/R injury. ROS and Ca^2+^ are both second messengers in cellular signaling pathways that regulate a variety of physiological and pathological processes. They are also involved in the occurrence and development of myocardial I/R injury. We previously revealed that F_2_ and classical calcium antagonists such as verapamil, diltiazem, and nifedipine could improve cardiac tissue or cardiomyocyte SOD activity during I/R or H/R, as well as reduce MDA content^15^. This indirectly suggests that they regulate cardiac tissue or cardiomyocyte ROS levels during I/R or H/R. Based on the result that abnormal ROS/JNK/Egr-1 signaling pathway is active in H9c2 cells during H/R, it is here speculated that the protective effects of F_2_ on myocardium might be related to its regulatory role in ROS/JNK/Egr-1 signaling.

The current study is the first to reveal that F_2_ could antagonize the H/R-induced increased ROS level, JNK activation, and Egr-1 expression in a dose dependent manner. Then, the dose of F_2_ (1 × 10^−6^ M) was selected based on previous reports of optimal protective effects^10^,^12^. Results showed that increasing ROS level in H9c2 cells using the ROS donor XO/HX could antagonize the F_2_-mediated inhibition of H/R-induced intracellular JNK activation and Egr-1 overexpression. Similarly, the JNK activator ANISO inhibited the F_2_-mediated downregulation of JNK activation and Egr-1 overexpression, suggesting that F_2_ downregulates Egr-1 by inhibiting ROS high-level and JNK activation. Therefore, we demonstrated that F_2_ can antagonize myocardial I/R injury via regulating ROS/JNK/Egr-1 signaling. These findings provide a theoretical basis for future studies assessing the application of F_2_ to treat of various diseases caused by oxidative stress.

## Conclusion

In summary, H/R leads to ROS/Egr-1 signaling pathway activation in H9c2 cells, and JNK activation mediates the signaling pathways between ROS and Egr-1. F_2_ regulates H/R-induced ROS/JNK/Egr-1 signaling, which might be an important mechanism by which it antagonizes myocardial I/R injury.

## Methods

### Reagent preparation

The H9c2 cell line was purchased from the Chinese Academy of Sciences (Beijing, China). Fetal bovine serum was obtained from Hyclone. F_2_ was dissolved in DMSO. The ROS scavenger EDA and NAC were purchased from Simcere Pharmaceuticals (Nanjing, China) and Sigma-Aldrich (United States), respectively. The ROS donors XO, HX, the JNK activator ANISO, and 2’,7’-dichlorofluorescein acetyl acetate (DCFH-DA) were purchased from Sigma-Aldrich. The JNK inhibitor SP600123 was purchased from Enzo Life Sciences (Switzerland). The p-JNK goat polyclonal antibody was purchased from Santa Cruz Biotechnology (United States), and rabbit JNK and Egr-1 monoclonal antibodies were purchased from Cell Signaling Technology (United States). Mouse β-actin antibody was purchased from Zhongshan Golden Bridge Biotechnology Co., Ltd. (Beijing, China). IRDye^TM^ 800CW goat anti-rabbit IgG and IRDye^TM^ 800CW goat anti-mouse IgG were purchased from United States LI-COR Inc. (United States). Horseradish peroxidase-labeled murine and rabbit secondary antibodies were purchased from Boster Biological Engineering Co., Ltd., (Wuhan, China). Hypoxia solution, which imitates the microenvironment of high-potassium, lactic acid accumulation and glucose depletion after myocardial ischemia *in vivo*, was prepared as described previously^32^: 137 mM NaCl, 12 mM KCl, 0.49 mM MgCl_2_ 6H_2_O, 0.9 mM of CaCl_2_, 4 mM HEPES, and 20 mM sodium lactate.

### Cell culture and establishment of H/R model

The H9c2 cells line was cultured in DMEM medium with 10% fetal bovine serum (FBS) at 37 °C with 5% CO_2_, and was synchronized treatment with medium containing 0.5% FBS for 12–24 h before every experiment. To induce hypoxia, which should be oxygen-free and nutrition-free for the sake of mimicking ischemia, H9c2 cells were cultured in hypoxic solution by saturation in advance with pure nitrogen for 30 min in order to expel the air, and then pure nitrogen gas was used to fill the culture vessels and hypoxia chamber. The cells placed in hypoxia chamber were cultured at 37 °C for 1, 2, or 3 h; After that, the hypoxia solution was then replaced with fresh oxygenated culture medium and the culture vessels were transferred into normoxic incubator (5% CO_2_ ) at 37 °C for 1 h of reoxygenation.

### Experimental Grouping

**Figure 7:**
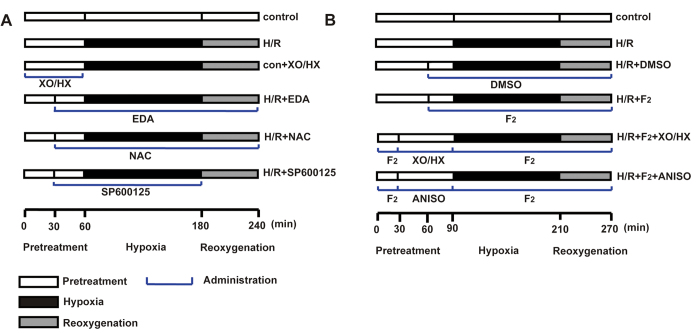
Protocol of the experimental grouping and treatments. **A**. Protocol used to investigate whether ROS /JNK/Egr-1 signaling occurs in H9c2 cells after H/R. **B**. Protocol used to investigate the effects of F_2_ on ROS/JNK/Egr-1 signaling in H9c2 cells after H/R.

H9c2 cells cultured for 2–3 days were grouped randomly as follows (Fig. 7): control, H/R, control + the ROS donor XO/HX (con + XO/HX), H/R + the ROS scavenger EDA (H/R + EDA), H/R + the ROS scavenger NAC (H/R + NAC), H/R + JNK inhibitor SP600125 (H/R + SP600125), H/R + different doses of F_2_, H/R + solvent (H/R + DMSO), H/R + F_2_ + XO/HX, and H/R + F_2 _+ JNK activator ANISO (H/R + F_2_ + ANISO). The control group was cultured with fresh medium for 2, 3, or 4 h before the experiments. The H/R group was cultured under nitrogen-saturated hypoxia at 37 °C for 1, 2, or 3 h, and was then cultured in fresh medium under normal conditions for 1 h. XO/HX (1 mU/ml/1.2 × 10^−4^ M, 3 mU/ml/3.6 × 10^−4^ M, 5 mU/ml /6.0 × 10^−4^ M), EDA (2 × 10^−6^ M, 2 × 10^−5^ M, 2 × 10^−4^ M), NAC (5 × 10^−4^ M, 2 × 10^−3^ M, 8 × 10^−3^ M), SP600125 (2 × 10^−5^ M), ANISO (10 ng/ml), and F_2_ (1 × 10^−7^ M, 1 × 10^−6^ M, 1 × 10^−5^ M) were prepared in normal medium (for preincubation), hypoxia solution, and/or reoxygenation medium.

### Western blotting

Western blotting was performed as described previously^10^. Briefly, total proteins were extracted and quantified. Next, 30–50 μg of each sample was separated using 10% SDS-PAGE gel electrophoresis (stacking gel 50 V, separating gel 100 V), and transferred to nitrocellulose membrane (100 V, 75 min). Membranes were then blocked and incubated with primary antibodies (Egr-1, p-JNK, JNK, and β-actin at 1:1,000, 1:200, 1:2,000, and 1:3,000 dilutions, respectively) followed by secondary antibodies (HRP-labeled rabbit anti-goat IgG, HRP-labeled goat anti-rabbit IgG, IRDye^TM^ 800CW goat anti-rabbit IgG, or IRDye^TM^ 800CW goat anti-mouse IgG at dilutions of 1:20,000, 1:30,000, 1:10,000, or 1:10,000, respectively); IRDye incubations were performed in the dark. HRP-labeled secondary antibodies were detected using chemiluminescence, and the grayscale of the protein bands was analyzed using Gel-pro Image Analysis Software (Media cybernetics, USA). IRDye-labeled secondary antibodies were analyzed using an Odyssey infrared imaging system (Odyssey LI-COR, USA). The ratio of p-JNK/JNK represented the activation of JNK, JNK/β-actin indicated the overall expression of JNK, and the ratio of Egr-1/β-actin reflected the expression of Egr-1.

### Detecting ROS levels in H9c2 cells using flow cytometry

Culture medium containing 0.5% FBS was used to treat cells for 12–24 h. Cells were trypsinized with 0.125% trypsin at room temperature, and collected in a 2 ml Eppendorf tube after the indicated treatments. The cells were then centrifuged for 5 min at 5 °C and 1000 rpm, and the supernatants were discarded. The pellets were washed with serum-free medium, centrifuged again, and the supernatants were discarded. The cell pellets were mixed with 1 ml serum-free medium containing a final concentration of 10 μM DCFH-DA probe, dispersed uniformly, and then incubated in the dark at 37 °C for 30 min with gentle shaking every 5 min to fully expose the cells to the probe. The mixtures were then centrifuged for 5 min at 25 °C and 1000 rpm, the supernatants were discarded, and the pellets were washed three times with PBS. Subsequently, the cell pellets were resuspended in 500 μl PBS, mixed well, and analyzed using a FACSCalibur flow cytometer (Becton Dickinson, USA). The green fluorescence emitted by ~10,000 cells was recorded at an excitation wavelength of 488 nm. Software WinMDI2.9 was used to analyze the mean fluorescence intensity (MFI).

### Statistical analysis

Data are presented as mean ± SEM. The significance of differences was determined using one-way ANOVA followed by Student-Newman-Keuls test. *P *< 0.05 was considered statistically significant.

## Additional Information

**How to cite this article**: Zhang, Y. *et al.* Effect of *N*-n-butyl haloperidol iodide on ROS/JNK/Egr-1 signaling in H9c2 cells after hypoxia/reoxygenation. *Sci. Rep.* **5**, 11809; doi: 10.1038/srep11809 (2015).

## References

[b1] Murphy E. & Steenbergen C. Mechanisms underlying acute protection from cardiac ischemia-reperfusion injury. Physiol Rev. 88, 581–609 (2008). 18391174 10.1152/physrev.00024.2007 PMC3199571

[b2] Pagel J. I. & Deindl E. Disease progression mediated by egr-1 associated signaling in response to oxidative stress. Int J Mol Sci. 13, 13104–13117 (2012). 23202940 10.3390/ijms131013104 PMC3497314

[b3] Minamino T. Cardioprotection from ischemia/reperfusion injury: basic and translational research. Circ J. 76, 1074–1082 (2012). 22504127 10.1253/circj.cj-12-0132

[b4] Madureira P. A. & Waisman D. M. Annexin A2: the importance of being redox sensitive. Int J Mol Sci. 14, 3568–3594 (2013). 23434659 10.3390/ijms14023568 PMC3588059

[b5] Cosentino-Gomes D., Rocco-Machado N. & Meyer-Fernandes J. R. Cell Signaling through Protein Kinase C Oxidation and Activation. Int J Mol Sci. 13, 10697–10721 (2012). 23109817 10.3390/ijms130910697 PMC3472709

[b6] Choudhury S., Panda P., Sahoo L. & Panda S. K. Reactive oxygen species signaling in plants under abiotic stress. Plant signal Beha. 8, e23681 (2013). 10.4161/psb.23681 PMC7030282 23425848

[b7] Kaufmann K. & Thiel G. Epidermal growth factor and thrombin induced proliferation of immortalized human keratinocytes is coupled to the synthesis of Egr-1, a zinc finger transcriptional regulator. J Cell Biochem. 85, 381–391 (2002). 11948693 10.1002/jcb.10145

[b8] Grohe C., Nouskas J., Vetter H. & Neyses L. Effects of nisoldipine on endothelin-1- and angiotensin II-induced immediate/early gene expression and protein synthesis in adult rat ventricular cardiomyocytes. J Cardiovas Pharmacol. 24, 13–16 (1994). 10.1097/00005344-199407000-00003 7521477

[b9] Yan S. F. *et al.* Egr-1, a master switch coordinating upregulation of divergent gene families underlying ischemic stress. Nat Med. 6, 1355–1361 (2000). 11100120 10.1038/82168

[b10] Zhang Y. *et al.* The protective effects of N-n-butyl haloperidol iodide on myocardial ischemia-reperfusion injury in rats by inhibiting Egr-1 overexpression. Cell Physiol Biochemi. 20, 639–648 (2007). 10.1159/000107547 17762190

[b11] Zhang Y. *et al.* The protective effect of Egr-1 antisense oligodeoxyribonucleotide on myocardial injury induced by ischemia-reperfusion and hypoxia-reoxygenation. Cell Physiol Biochemi. 22, 645–652 (2008). 10.1159/000185548 19088446

[b12] Wang J. Z. *et al.* N-n-Butyl haloperidol iodide protects against hypoxia/reoxygenation-induced cardiomyocyte injury by modulating protein kinase C activity. Biochem Pharmacol. 79, 1428–1436 (2010). 20105432 10.1016/j.bcp.2010.01.021

[b13] Gao F. F. *et al.* Cardiac electrophysiological and antiarrhythmic effects of N-n-butyl haloperidol iodide. Cell Physiol Biochemi. 25, 433–442 (2010). 10.1159/000303048 20332624

[b14] Zhang Y. *et al.* N-n-butyl haloperidol iodide ameliorates cardiomyocytes hypoxia/reoxygenation injury by extracellular calcium-dependent and -independent mechanisms. Oxid Med Cell Longevi. 2013, 912310 (2013). 10.1155/2013/912310 PMC3857550 24392181

[b15] Huang Z. *et al.* Egr-1, the potential target of calcium channel blockers in cardioprotection with ischemia/reperfusion injury in rats. Cell Physiol Biochemi. 24, 17–24 (2009). 10.1159/000227809 19590189

[b16] Lamb R. E. & Goldstein B. J. Modulating an oxidative-inflammatory cascade: potential new treatment strategy for improving glucose metabolism, insulin resistance, and vascular function. Int J Clin Pract. 62, 1087–1095 (2008). 18489578 10.1111/j.1742-1241.2008.01789.x PMC2440526

[b17] Mkaddem S. B., Bens M. & Vandewalle A. Differential activation of Toll-like receptor-mediated apoptosis induced by hypoxia. Oncotarget 1, 741–750 (2010). 21321383 10.18632/oncotarget.209 PMC3157738

[b18] Sun K. H., Lee H. G., Smith M. A. & Shah K. Direct and indirect roles of cyclin-dependent kinase 5 as an upstream regulator in the c-Jun NH2-terminal kinase cascade: relevance to neurotoxic insults in Alzheimer’s disease. Mol Biol Cell. 20, 4611–4619 (2009). 19776350 10.1091/mbc.E09-05-0433 PMC2770948

[b19] Saben J. *et al.* Early growth response protein-1 mediates lipotoxicity-associated placental inflammation: role in maternal obesity. Am J Physiol Endocrinol Metab. 305, E1–14 (2013). 23632636 10.1152/ajpendo.00076.2013 PMC4116409

[b20] Choi S., Na H. Y., Kim J. A., Cho S. E. & Suh S. H. Contradictory Effects of Superoxide and Hydrogen Peroxide on KCa3.1 in Human Endothelial Cells. Korean J Physiol Pharmacol. 17, 181–187 (2013). 23776393 10.4196/kjpp.2013.17.3.181 PMC3682077

[b21] Kelley E. E. *et al.* Hydrogen peroxide is the major oxidant product of xanthine oxidase. Free Radic Biol Med. 48, 493–498 (2010). 19941951 10.1016/j.freeradbiomed.2009.11.012 PMC2848256

[b22] Han J., Shuvaev V. V. & Muzykantov V. R. Catalase and superoxide dismutase conjugated with platelet-endothelial cell adhesion molecule antibody distinctly alleviate abnormal endothelial permeability caused by exogenous reactive oxygen species and vascular endothelial growth factor. J Pharmacol Exp Ther. 338, 82–91 (2011). 21474567 10.1124/jpet.111.180620 PMC3126647

[b23] Jeong S. H. *et al.* ZnO nanoparticles induce TNF-alpha expression via ROS-ERK-Egr-1 pathway in human keratinocytes. J Dermatol Sci. 72, 263–273 (2013). 24001789 10.1016/j.jdermsci.2013.08.002

[b24] Han M. H. *et al.* Apoptosis induction of human bladder cancer cells by sanguinarine through reactive oxygen species-mediated up-regulation of early growth response gene-1. PloS one 8, e63425 (2013). 23717422 10.1371/journal.pone.0063425 PMC3661671

[b25] Han M. H., Kim G. Y., Yoo Y. H. & Choi Y. H. Sanguinarine induces apoptosis in human colorectal cancer HCT-116 cells through ROS-mediated Egr-1 activation and mitochondrial dysfunction. Toxicol Lett. 220, 157–166 (2013). 23660334 10.1016/j.toxlet.2013.04.020

[b26] Jeon H. M. *et al.* Early growth response 1 regulates glucose deprivation-induced necrosis. Oncol Rep. 29, 669–675 (2013). 23152075 10.3892/or.2012.2134 PMC3583586

[b27] Aggeli I. K., Beis I. & Gaitanaki C. ERKs and JNKs mediate hydrogen peroxide-induced Egr-1 expression and nuclear accumulation in H9c2 cells. Physiol Res. 59, 443–454 (2010). 19681663 10.33549/physiolres.931806

[b28] Hartney T. *et al.* Xanthine oxidase-derived ROS upregulate Egr-1 via ERK1/2 in PA smooth muscle cells; model to test impact of extracellular ROS in chronic hypoxia. PloS one 6, e27531 (2011). 22140445 10.1371/journal.pone.0027531 PMC3225357

[b29] Nozik-Grayck E. *et al.* Lung EC-SOD overexpression attenuates hypoxic induction of Egr-1 and chronic hypoxic pulmonary vascular remodeling. Am J Physiol Lung cell Mol Physiol. 295, L422–430 (2008). 18599502 10.1152/ajplung.90293.2008 PMC2536799

[b30] Mo Y. *et al.* Combination effects of cigarette smoke extract and ambient ultrafine particles on endothelial cells. Toxicol In vitro. 26, 295–303 (2012). 22178768 10.1016/j.tiv.2011.12.001 PMC3273600

[b31] Thiel G., Mayer S. I., Muller I., Stefano L. & Rossler O. G. Egr-1-A Ca(2+)-regulated transcription factor. Cell calcium 47, 397–403 (2010). 20303171 10.1016/j.ceca.2010.02.005

[b32] Zhang Y. M. *et al.* Effects of N-n-butyl haloperidol iodide on myocardial ischemia/reperfusion injury and Egr-1 expression in rat. Acta Biochim Biophys Sin. 38, 435–441 (2006). 16761102 10.1111/j.1745-7270.2006.00180.x

